# Acceptability of the Cytosponge procedure for detecting Barrett's oesophagus: a qualitative study

**DOI:** 10.1136/bmjopen-2016-013901

**Published:** 2017-03-01

**Authors:** Madeleine Freeman, Judith Offman, Fiona M Walter, Peter Sasieni, Samuel G Smith

**Affiliations:** 1 Wolfson Institute of Preventive Medicine, Queen Mary University of London, London, UK; 2 Institute of Epidemiology and Healthcare, University College London, London, UK; 3 Primary Care Unit, Department of Public Health & Primary Care, University of Cambridge, Cambridge, UK; 4 Leeds Institute of Health Sciences, University of Leeds, Leeds, UK

**Keywords:** Cytosponge, Barrett’s oesophagus, Oesophageal adenocarcinoma, Acceptability, QUALITATIVE RESEARCH, Diagnosis

## Abstract

**Objective:**

To investigate the acceptability of the Cytosponge, a novel sampling device to detect Barrett's oesophagus (BE), a precursor to oesophageal adenocarcinoma (EAC), among people with risk factors for this condition.

**Design:**

A qualitative study using semistructured interviews and focus group discussions. Data were explored by three researchers using thematic analysis.

**Setting:**

Community setting in London, UK.

**Participants:**

A recruitment company identified 33 adults (17 men, 16 women) aged 50–69 years with gastro-oesophageal reflux disease (GERD), a risk factor for BE. The majority of participants were white British (73%). The focus groups were stratified by gender and education. 10 individuals were interviewed and 23 participated in four focus groups.

**Results:**

3 key themes emerged from the data: the anticipated physical experience, preferences for the content of information materials and comparisons with the current gold-standard test. Overall acceptability was high, but there was initial concern about the physical experience of taking the test, including swallowing and extracting the Cytosponge. These worries were reduced after handling the device and a video demonstration of the procedure. Knowledge of the relationship between GERD, BE and EAC was poor, and some suggested they would prefer not to know about the link when being offered the Cytosponge. Participants perceived the Cytosponge to be more comfortable, practical and economical than endoscopy.

**Conclusions:**

These qualitative data suggest the Cytosponge was acceptable to the majority of participants with risk factors for BE, and could be used as a first-line test to investigate GERD symptoms. Concerns about the physical experience of the test were alleviated through multimedia resources. The development of patient information materials is an important next step to ensuring patients are adequately informed and reassured about the procedure. Patient stakeholders should be involved in this process to ensure their concerns and preferences are considered.

**Trial registration number:**

ISRCTN68382401; pre-results.

**Strengths and limitations of this study**

- This is the first study to qualitatively explore the acceptability of the Cytosponge test among members of the public who may be at increased risk for Barrett's oesophagus.
- The sample was diverse in terms of gender and educational background.
- Participants were recruited using a market research company, so results may represent the views of particularly motivated individuals and should be carefully generalised to the wider patient population.
- Participants were not attending a medical appointment, so their attitudes towards the Cytosponge were hypothetical and may not reflect their opinions in a clinical setting.
- Quantitative data on the acceptability of the Cytosponge and its influence on test uptake were not assessed, but this has been previously examined in a clinical setting.

## Introduction

In the UK, 8784 individuals were diagnosed with oesophageal cancer in 2013.^1^ The majority of oesophageal cancers occur as either squamous cell carcinomas or adenocarcinomas (EACs), two cancers with different aetiologies.^2^ EAC mainly originates from Barrett's mucosa. Barrett's oesophagus (BE), a precursor of EAC, is a complication of chronic gastro-oesophageal reflux disease (GERD), where the stomach contents rise into the oesophagus often due to a malfunctioning lower oesophageal sphincter muscle. The reflux of acid and bile damages the squamous cells lining the oesophagus, which are then repaired through metaplastic columnar epithelium instead of the regeneration of more squamous cells.^3^ The risk of malignant progression is low, with an estimated annual incidence of EAC of 0.1–0.5% in individuals with BE.^4–6^

Risk factors for BE include male gender, Caucasian ethnicity, over 50 years of age, and having a family history of BE. Lifestyle factors include being overweight or obese, high abdominal fat, smoking and drinking alcohol.^7–9^ US and UK data suggest approximately one in five adults experience GERD in their lifetime,^10^ and 5–20% of adults with GERD are affected by BE.^11^ ^12^ BE is an asymptomatic condition that is usually only discovered during endoscopy performed for evaluation of GERD symptoms. The majority of EACs, however, present de novo without a prior diagnosis of BE,^13^ and symptomatically detected EACs are normally advanced. In the UK, the overall EAC 5-year survival rate is as low as 19%.^14^

Identifying patients with undiagnosed BE may reduce mortality; however, routinely investigating all patients with dyspepsia and GERD using endoscopy would be time and resource intensive, and not feasible in the National Health Service (NHS).^14^ The Cytosponge is a non-endoscopic, ingestible oesophageal sampling device, which is both minimally-invasive and potentially cost-effective.^15^ ^16^ It can be administered in various settings including primary care. Patients with a history of GERD symptoms are asked to swallow a gelatine capsule on a string, which dissolves in the stomach. As the capsule dissolves a small sponge expands and is pulled out of the mouth, collecting cells from the oesophagus in the process. The Cytosponge takes 5 min to administer. Immunohistochemistry is performed on the sponge to detect Trefoil Factor 3 (TFF3), a biomarker for BE.

The acceptability and accuracy of the Cytosponge-TFF3 test for screening for BE has been tested in several studies, including the Barrett's Oesophagus Trial 1 (BEST1), a cohort study in patients with previous prescriptions for acid suppressants in a UK primary care setting,^16^ and BEST2, a case–control study in patients with GERD with or without BE in a UK hospital setting.^17^ It was shown to have high sensitivity (73.3% and 79.9%, respectively) and specificity (93.8% and 92.4%, respectively) for detecting BE. Moreover, over 93% of patients successfully swallowed the Cytosponge and 82% reported low levels of anxiety before the test, suggesting high acceptability. We are currently in the process of setting up a randomised control trial, BEST3, to evaluate if the Cytosponge-TFF3 test leads to an increase in the number of patients diagnosed with BE in primary care, and to gain an in-depth understanding of the associated health economics. The acceptability of the procedure and preferences regarding the presentation of information on the test are an important part of this process, and has not been investigated in any detail until now.

To ensure information on the procedure will be presented to BEST3 participants in the most accessible manner, and to encourage rapid implementation in clinical practice at a later stage, public perceptions of the procedure need to be explored in greater depth. The aim of this study was to obtain a detailed understanding of the acceptability of the Cytosponge-TFF3 test in a sample of individuals with GERD. This population was chosen because they reflect the population to which the test will be offered as part of the BEST3 trial and if it is implemented in routine primary care.

## Methods

### Participants

The study was advertised to adults aged 50–69 years by a recruitment company email, and respondents were screened on the telephone for eligibility. This age group was selected as this population will be eligible for the Cytosponge test within the BEST3 trial. GERD severity was assessed using the GERD impact scale.^18^ Individuals were eligible if they experienced one of five GERD symptoms (eg, heartburn), or used GERD medications, ‘sometimes’, ‘often’ or ‘daily’. Individuals with a medical occupation or previous cancer diagnosis were excluded. The recruitment company were provided with quotas to ensure at least half the sample were men and half did not have an academic degree. Respondents with an academic degree were classified as having a high level of education. The number of people who were approached or refused is not known.

The first 10 eligible respondents were invited to participate in a one-to-one interview with a researcher (MF). The interviews were used to inform the focus group presentation. After 10 interviews were completed four focus groups were arranged. The focus groups were stratified by gender (male and female) and education (high vs basic). Participants were recruited until data saturation was reached and no new themes emerged from the focus groups or interviews.^19^

### Procedure

Semi-structured interviews are an effective method for obtaining detailed opinions of an isolated individual, whereas focus groups generate discussion from multiple perspectives. A combination of both face-to-face interviews and focus group discussions were used for this study to explore how individuals perceived the Cytosponge test when they were alone and in a group setting. Interviews and focus group discussions took place in meeting rooms at the Wolfson Institute for Preventive Medicine, Queen Mary, University of London. Written consent was obtained at the beginning of the interview or focus group by a female research assistant trained in qualitative research (MF, MSc Health Psychology). The interviewer had not met any participant prior to the study, and participants were given little information beyond her research interests. MF had no personal interest in oesophageal cancer research that would influence her behaviour towards the participants. No other person was present within the interview or focus group. A demographic and lifestyle questionnaire was completed prior to starting. This assessed GERD severity and management with the GERD impact scale,^18^ as well as experience of endoscopy, age, ethnicity, education and smoking status. All interviews and focus group discussions were audio recorded. Participants were compensated £40 for their time and travel. The consolidated criterion for reporting qualitative research was used.^20^

### Interviews

The interview schedule was designed by the research team and tested with three adults aged over 50 years to ensure participants could follow all questions in the different sections. In response to these test interviews background sections were expanded or simplified where necessary and a small number of questions about the Cytosponge were added. These interviews, however, were not recorded or included in the analysis.

**Figure 1:**
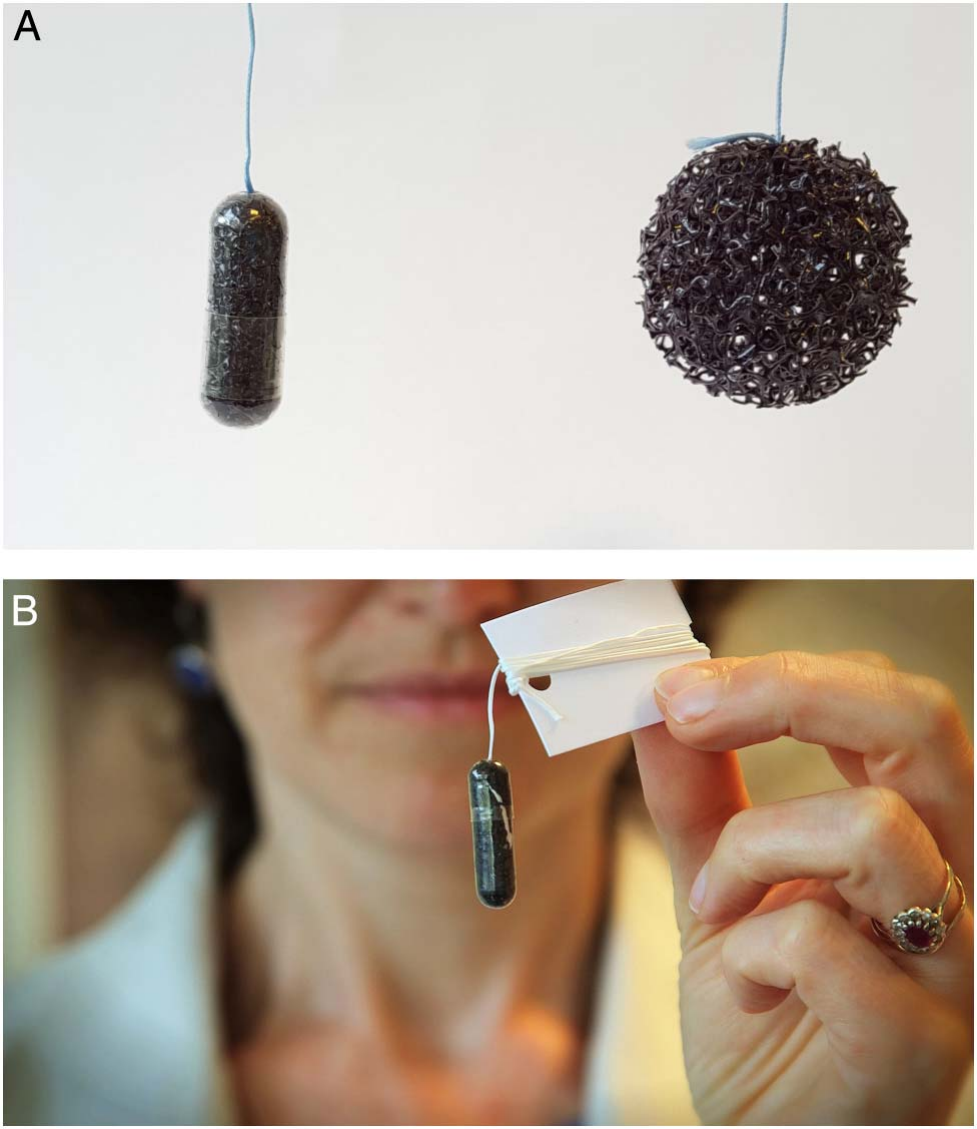
Cytosponge images used in interviews and focus groups. (A) Cytosponge in gelatine capsule (left) and expanded (right); (B) Cytosponge in gelatine capsule attached to string and cardboard as would be used for the Cytosponge procedure.

The interview schedule had six sections, also see online supplementary document 1: participants' understanding of GERD and its association with BE and EAC, their first impressions of the Cytosponge test, their perceptions of the BEST3 trial, their attitudes towards endoscopy, the potential impact of cancer prevention on their decision-making (table 1). At prespecified points of the interview, photographs of the Cytosponge were shown (figure 1), as well as physical examples of the device and a video of a patient undergoing the Cytosponge test.

**Table 1:** Summary of interview schedule

Summary of interview schedule	
Understanding of GERD	Discussion about GERD and other symptoms Causes, risk factors, consequences, self-management, treatments, help-seeking
Links between conditions	Awareness of BE and explanation of link with GERD and oesophageal cancer
Cytosponge test	Verbal and photographic description of Cytosponge First impressions of Cytosponge based on images Willingness to have test, preferences for terminology used, aesthetics of Cytosponge, barriers to use Show Cytosponge (capsule and sponge) Impressions after demonstration Video of Cytosponge test https://www.youtube.com/watch?v=s7X9z6qlNUI Impressions after video
BEST3 trial	Willingness to take part in trial (if offered) Perceptions of test accuracy, risks, practicalities of trial, information preferences
Endoscopy	Diagram of endoscopy Experiences of endoscopy Test preferences: Cytosponge vs endoscopy
Cancer prevention	Potential impact of new cancer prevention methods on willingness to have the Cytosponge

BE, Barrett's oesophagus; BEST3, Barrett's Oesophagus Trial 3; GERD, gastro-oesophageal reflux disease.

10.1136/bmjopen-2016-013901.supp1 supplementary document

### Focus groups

A presentation was created by the research team. This followed a similar structure to the interview schedule, although questions relating to the topic of ‘Cancer Prevention’ were removed, as participants had difficulty answering these in the interviews. The presentation contained more visual cues than the interview and participants could read the text, which was in bullet-point format, and projected onto a screen (see online supplementary document 2). As with the interviews, photographs (figure 1), physical examples and a video of the Cytosponge test were shown at prespecified points. Each focus group was moderated by MF who was supported by a second member of the research team (SS or AM). Notes were taken by the second researcher.

10.1136/bmjopen-2016-013901.supp2 supplementary document

### Analysis

Interviews and focus group recordings were transcribed verbatim by MF. Thematic analysis, a technique for identifying patterns (themes) within qualitative data was used for the analysis.^21^ Transcripts were read by MF, SS and JO to familiarise themselves with the data. The transcripts were then coded by MF to identify initial themes in the data. Using these themes, MF and SS created an analytical model on which to base the remaining analysis. Themes within the model were revised by MF, SS and JO iteratively throughout the analysis. Representative quotes were chosen to illustrate the themes identified within the data set. Consideration was given to ensure all sociodemographic groups were adequately represented in the quotations. It was agreed by the three researchers involved in the analysis that data saturation had been reached, and no further interviews or focus group discussions were necessary. Participants were not given an opportunity to review the transcripts. Microsoft Excel was used to support the analysis.

## Results

### Sample characteristics

Thirty-three individuals participated in the study, 16 women and 17 men (table 2). Ages ranged from 50 to 69 years, with a mean age of 57 years. The majority of participants were white British (73%), approximately half of them were educated to degree level (52%), and 45% of the sample had experience of endoscopy. Sampling of roughly equal numbers of participants with previous experience with endoscopy occurred by chance rather than through purposive sampling. Ten individuals were interviewed and 23 took part in focus groups. Interviews ranged in length between 26 and 57 min, and focus group discussions between 64 and 86 min, with participants spending the longest discussing their personal heartburn experience and their impression of the Cytosponge.

**Table 2:** Demographic characteristics for study participants (n=33)

	n (%)
Gender	
Female	16 (48.5)
Male	17 (51.5)
Age (years)	
50–59	23 (69.7)
60–69	10 (30.3)
Highest level of education	
O-Levels	10 (30.3)
A-Levels	6 (18.2)
Degree	17 (51.5)
Ethnicity	
White British	24 (72.7)
White Irish	1 (3.0)
White and Asian	1 (3.0)
Caribbean	1 (3.0)
Missing	6 (18.2)
Smoking status	
Smoker	6 (18.2)
Ex-smoker	9 (27.3)
Never smoked	12 (36.4)
Missing	6 (18.2)
Previous experience of endoscopy	
Yes	15 (45.5)
No	18 (54.5)

Participants expressed enthusiasm about the Cytosponge, with most indicating they would take the test if invited. The three major themes identified were the anticipated physical experience, information preferences and comparisons with endoscopy. Subthemes are presented below, along with representative quotes.

### Anticipated physical experience

Individuals discussed their anticipated physical experience of the Cytosponge test. This included swallowing the Cytosponge and extracting it. The anticipated physical experience appeared to be shaped by both handling examples of the capsule and sponge, and watching the video of the test being carried out.

#### Swallowing the sponge

Based on initial images and verbal descriptions most individuals imagined the capsule to be bigger than its actual size. People expressed surprise and felt reassured after handling a Cytosponge. It was generally felt that the act of swallowing the Cytosponge capsule would not be problematic, because it was of a similar size and shape to other tablets they take regularly.

It's smaller than I imagined … The capsule is just like a normal vitamin capsule, so that's perfectly fine (Participant 4, Focus Group 3, female, age 56, high education)

A few people were concerned about swallowing the string attached to the capsule.

It's just the string following it down I think I might have a problem with. I’ve never actually swallowed a ball of string (Participant 4, Focus Group 2, male, age 51, basic education)

#### Extracting the sponge

After feeling an expanded Cytosponge in the demonstration, participants said it was rougher than they expected, with many comparing it to a ‘Brillo pad’. As a result, there was concern that an expanded Cytosponge would damage their oesophagus when it was extracted.

You’re going to feel it, it's going to be as if somebody's got their fingers and scratching the inside of your windpipe (Participant 6, Focus Group 2, male, age 56, basic education)

Some people were worried they would gag or even vomit when the Cytosponge was withdrawn from the stomach.

I’d be frightened that I’d throw up. I’ll be honest (Participant 5, Focus Group 4, female, age 55, basic education)

A consistent concern was the possibility of the string breaking and the Cytosponge getting stuck in the oesophagus or stomach.

What if it got stuck? Because you know sometimes when a sweet goes down the wrong way…and it gets stuck? That is scary (Participant 2, Focus Group 3, female, age 52, high education)

#### Video reassurance

After watching the video, individuals were more positive about the Cytosponge test compared with their initial reactions. The main reason for this attitude shift was because the extraction of the sponge was considerably quicker than expected.

In my mind I was thinking of you know [removing the sponge would be like] catching a fish (Participant 5, Focus Group 2, male, age 50, basic education)

Additionally, the ease with which the patient swallowed the Cytosponge was commented on. The fact the patient did not gag during its extraction was considered comforting.

He didn’t show any gagging or anything. He was absolutely fine (LK, Interview, female, age 59, basic education)

Finally, people commented that seeing the patient in the video looking relaxed put them at ease.

He looked very relaxed, surprisingly… I think I'm sort of happier now, after watching the video (Participant 6, Focus Group 1, male, age 60, high education)

### Information preferences

Individuals differed in their preferences for what information they would receive if they were asked to do the Cyosponge test. This included the amount of information they were given on the link between GERD, BE and EAC, on the Cytosponge test itself, whether they were shown an expanded Cytosponge before taking the test, and the optimal timing for informing patients about endoscopy referral. People also discussed their preferred format for communicating information.

#### Information on cancer link

Most participants had no or low awareness of the relationship between GERD, BE and EAC.

I’ve never taken it [GERD] to the next step in my mind (Participant 2, Focus Group 3, female, age 52, high education)

Some people said they would want to be told GERD can lead to EAC before they took the Cytosponge test, while others said they would not want to know about the association. Some perceived that discussing a link with cancer may frighten patients and reduce uptake.

If you’re reading something with ‘cancer’, you’re frightening them anyway… You say cancer, people won't take the pill (Participant 1, Focus Group 2, male, age 58, basic education)

#### Information on Cytosponge test

Most participants felt the amount of information provided about the Cytosponge test during the interview or focus group was sufficient for them to decide whether they would have the Cytosponge test.

I'd want to know everything you told us here [referring to earlier verbal descriptions, sample demonstrations and the video]. … What it does, how it does it, why you're doing it? (Participant 4, Focus Group 2, male, age 51, basic education)

In addition, some people wanted to be informed about other details for example whether the test had to be performed on an empty stomach. There was also demand for information on side effects, even though they felt that those would probably be unlikely or mild.

If there was any side effect, whether it has to be done on an empty stomach, like, you know, should it be done in the morning? (Participant 5, Focus Group 4, female, age 55, basic education)

Other information people felt should be included regarded timings, specifically how long the test takes in total, how long the sponge stays in the stomach and the time it takes to receive results.

Yeah, so what happens? Once it goes to the lab, how long will it take before you find out whether you've got it? (Participant 6, Focus Group 3, female, age 54, high education)

Furthermore, individuals felt that information on the accuracy of the test should be included.

I'd want to know how, like I say, I'd want to know how accurate it is as well. There's no point doing it if it's not accurate (Participant 4, Focus Group 2, male, age 51, basic education)

#### Demonstration of Cytosponge test

During the demonstration, some participants expressed surprise at the appearance of the Cytosponge after the capsule had dissolved and it had expanded. They felt that prospective patients would be unnerved by its change in appearance if they had not been shown how the Cytosponge looks after it has been extracted before the test.

If they don’t tell you, you’re going to be in for a fright when you pull it out of your throat (Participant 4, Focus Group 2, male, age 51, basic education)

One person conceded this may not be necessary, and that a similar approach would not be taken with other medical procedures.

They wouldn’t do that with any other test, would they? They wouldn’t let you feel the needle before they took your blood (Participant 2, Focus Group 3, female, age 52, high education)

Another approach would be to offer each patient a choice.

I think it would be nice to be offered the choice. Maybe be asked ‘would you like to see how it looks? (Participant 5, Focus Group 2, male, age 50, basic education)

#### Test outcomes

There was debate over whether or not one should be told before taking the Cytosponge test that a positive result will necessitate further investigation. Some people insisted they would expect to be fully informed about the whole process at the outset, and compared it with the existing NHS screening programmes.

I imagine it's just like NHS screening actually, when you have a mammogram you’re actually told what the programme is (Participant 5, Focus Group 3, female, age 60, high education)

Others felt they would prefer to be told this information in a step by step approach, only being informed about the need for endoscopy after they have received a positive result. This preference was perceived to prevent unnecessary anxiety.

I think that just having one test is scary enough for someone, thinking they might have cancer. So just stick to that test, and when the results come back, then they can be told what the next step is (LK, Interview, female, age 59, basic education)

#### Information format

Peoples' preferences for the modality in which information about the Cytosponge test was delivered varied. Suggestions included pictures, a leaflet, a website and a video. The preferred modality was an information leaflet with a website link to the video of the test being carried out.

A link within the leaflet that you can actually go and see that video of the guy. I think that's really important (Participant 2, Focus Group 1, male, age 53, high education)

A video was perceived by some individuals as being easier to understand than written information.

Sometimes if it's late at night and my brain has, sort of, had enough for the day, I’d much rather just watch a little clip, you know I can get to see, and I can listen, I don’t have to read… reading is something you really have to really concentrate. So, the clip is almost doing it all for you (RH, Interview, female, age 54, basic education)

One participant mentioned that she would also like to see a follow-up video of the patient so that she would know what to expect after the test had been performed.

I would like to see him saying how he feels the next day… just another follow-up video…you know, ‘I’ve got a really bad sore throat, and, I feel a bit raw down there (AL, Interview, female, age 51, high education)

### Comparisons with endoscopy

While the Cytosponge test is not intended to replace endoscopy, it was felt that the new device was preferable physically, practically and economically.

#### Discomfort

Fifteen participants had previously undergone endoscopies for their heartburn, and almost all reported an unpleasant experience. By comparison, the Cytosponge test was perceived to be a more comfortable procedure.

The last [endoscopy] the nurse had to practically sit on me to stop me moving, because it was so unpleasant. And I was just regurgitating all this sort of yellow stuff, it was like something from a horror film (GC, Interview, male, age 51, high education)

[The Cytosponge] is such an innocuous test. You swallow a pill, you pull it out, two weeks later, done. I can’t see anybody saying no to that (IF, Interview, male, age 55, high education)

#### Practical factors

Individuals were enthusiastic that the Cytosponge test would be a quicker procedure than endoscopy, could be carried out by their general practitioner, and would not require an anaesthetic.

It's quicker, my doctor can do it and there's no messing around, no hospital appointments (AL, Interview, female, age 51, high education)

The fact that people would be able to resume their everyday activities immediately after the procedure was also seen as a benefit.

You can walk out of the doctor's surgery… you can get on with your everyday life (Participant 5, Focus Group 4, female, age 55, basic education)

#### Economic factors

A minority of people considered the superior cost-effectiveness of the Cytosponge test, and the benefits this would have for the healthcare system.

That's going to be an awful lot cheaper to do than an endoscopy at an hour a go with a gastroenterologist (KP, Interview, female, age 58, basic education)

## Discussion

In this sample of UK adults with GERD, the Cytosponge test was considered to be an acceptable and simple test to detect BE. Three key themes emerged from the data, including the anticipated physical experience of the test, preferences for the format and content of information, and comparisons with the current gold-standard test. These data can be used to inform the development of information materials and assist clinicians communicating with the public about the test.

Initial reactions to the Cytosponge test focused on the anticipated physical experience. Common concerns included worries about swallowing the capsule and string, the texture of the sponge once the capsule had dissolved, and the potential for the device to break and become stuck. Despite these worries, nearly all participants were reassured by seeing examples of the device and a video of a previous patient undergoing the procedure. The observation that participants felt more inclined to take the Cytosponge test after watching the video demonstration reflects previous research into the use of multimedia to promote health behaviours,^22^ including screening attendance.^23^ Video demonstration of the test could be an important communicative approach to reduce test anxiety and promote informed uptake.

Participants' information preferences varied. Opinions differed on how much information should be presented on the link between GERD, BE and EAC, test outcomes, and whether an example of an expanded Cytosponge should be shown. The content of health information materials is generally driven by the ethical and legal responsibility to promote informed decision-making.^24^ The preference of some patients to receive limited information therefore creates a tension between respecting public opinion, while also acting ethically and responsibly. The volume of questions asked by participants in the study and the variety of preferences suggests care should be taken during the design and evaluation of information resources. Ensuring the perspective of patient stakeholders is considered during this process may help to accommodate patient preferences. Evidence-based techniques and strategies for promoting comprehension should also be used to develop such resources.^25^ ^26^

Overall, participants felt that the Cytosponge would be preferable for potential patients compared with the current gold-standard test, endoscopy. The device was considered superior in terms of the physical experience, the practical simplicity and the potential for economic savings to the healthcare service. Initial concerns about the device notwithstanding, public enthusiasm for the Cytosponge test is encouraging. On the basis of this initial acceptability study, recruitment of patients to the BEST3 trial and subsequently implementing the device within a primary care setting is unlikely to face insurmountable barriers from the public.

Our study is the first to explore in-depth attitudes towards the Cytosponge test in a sample of adults with GERD. The use of interviews and focus groups provided a rich data set consisting of opinions expressed individually as well as in a group environment. The sample was diverse in terms of educational background and gender, allowing multiple perspectives to be considered. The study did have limitations. The sample was recruited using a market research company, and respondents may have been particularly motivated or interested in the topic. We did not collect quantitative data on acceptability or attitudes towards the test. Finally, the participants were not attending a medical appointment and therefore their attitudes towards the Cytosponge test were hypothetical, and may not reflect the opinions of patients in a clinical setting.

This study has several implications for clinical practice. Both for BEST3 and if the Cytosponge test is implemented in routine care it will be important for information materials and health professionals to reassure patients about the procedure. In particular, concerns about the size of the device, the string breaking and the physical sensation on the throat when the device is withdrawn should be addressed. The speed with which the procedure can be completed should be emphasised to prevent exaggerated assumptions about a lengthy extraction. The study showed individuals hold strong preferences regarding the amount and format of information they are given. Consequently, health professionals should consider eliciting information preferences during the consultation. Finally, we demonstrated a need to raise awareness of the link between GERD, BE and EAC. Clinicians should not assume that patients are aware of the associations, and should carefully explain the context when discussing why recurrent GERD symptoms should be investigated.

## Conclusion

This qualitative study revealed adults with GERD considered the Cytosponge test to be acceptable physically, practically and economically, as well as being preferred to endoscopy. If the device is implemented in routine clinical practice, it will be necessary to reassure patients about the Cytosponge and provide materials that meet the information needs of the patient group.
