# Supplementary material for: Acceptability of the Cytosponge procedure for detecting Barrett's oesophagus: a qualitative study
Source: BMJ Open. 2017 Mar 1;7(3):e013901. doi: 10.1136/bmjopen-2016-013901 (PMC5353314; doi:10.1136/bmjopen-2016-013901)
Supplement: supplementary document [file bmjopen-2016-013901supp1.pdf]

Supplementary Document 1: Interview Schedule

Changes made after the pilot interviews are highlighted in blue.

Introduction

Thank you very much for agreeing to take part in this study. Before we continue, I'd like to remind you of your rights as a participant. If at any point you would like to stop, or if you do not want to answer a question, please say so. You can also withdraw from the study at any point without giving a reason, and you will not experience a penalty for doing so. Anything you say to me today will be kept confidential, and if you are quoted in any of our reports, your name will not be mentioned and you will be given a false name (pseudonym). If you would like any further information about anything that we discuss today, you can contact either myself or the charities on the information sheet. Are you happy to continue?

- Please could you complete this questionnaire?
  (Insert demographic questionnaire)

- I am now going to start recording the interview. Are you happy for me to do this?
  (Begin recording)

1) Understandings of Heartburn

- So first of all, please could you tell me about your heartburn? Please go into as much detail as possible.

Prompts:

- *What do you think causes your heartburn?*
- *What do you think increases someone's chances of getting heartburn?*
- *Do you think your heartburn will have any serious consequences? What?*
- *Is there anything that you do when you have heartburn to make yourself feel better?*
- *Have you ever visited a doctor about your heartburn?*
- *If so, what made you go?*
- *Do you take any treatment for your heartburn? What?*

2) Link between Heartburn, Barrett's Oesophagus and Oesophageal cancer

Now I would like to give you some more information about why we are doing this study. Many people experience heartburn and it can usually can be treated with medication and lifestyle changes, for example avoiding eating heavy meals late at night. However, some people who have regular heartburn develop a condition called Barrett's Oesophagus. This is when the cells in the food pipe change shape. About 4 in 100 people with regular heartburn develop Barrett's Oesophagus.

- Have you ever heard of this condition before?
  (If yes ask them to go into detail about what they know)

The reason we are interested in Barrett's Oesophagus is because people with Barrett's have a slightly higher chance of developing Oesophageal cancer. Oesophageal cancer is still very rare though (1:200) and most people who have Barrett's Oesophagus will not develop it. Unfortunately, at the moment, most cases of this cancer are diagnosed late, and cannot be treated very well. We therefor are interested in using Barrett's oesophagus to help us diagnose cancers earlier or prevent it from developing. Even though we cannot treat Barrett's itself at the moment, we can use it as a way of identifying people who have a higher change of getting oesophageal cancer. So if Barrett's Oesophagus is found, that person can be monitored regularly (every one or two years) to find cancer earlier and be offered treatment earlier if cancer occurs.

3) Impressions of the Cytosponge

Scientists at the University of Cambridge have developed a new test to identify Barrett's Oesophagus called the Cytosponge test. This is what it looks like…

(Show pictures/ sample of the cytosponge)

The cytosponge is a small (2cm) capsule on a string which you swallow. After the cytosponge has been swallowed, it sits in the stomach for three or four minutes and expands. The sponge is pulled back up out of your mouth using the string attached. The whole process takes up to five minutes and can be carried out by a nurse or doctor. The sponge picks up about half a million tiny cells from your oesophagus and these are sent to the laboratory to look for signs of Barrett's Oesophagus.

(To do this we stain the cells for a molecular marker called TFF3 (Trefoil Factor 3), which only occurs in cells from a Barrett's but not a non-Barrett's Oesophagus. We can stain the cells we have collected using the cytosponge and then look under the microscope to distinguish between cells from an oesophagus with Barrett's compared to one without.) *only if someone asks about it.*

After seeing and hearing this information, what are your first impressions of this test?

Prompts:

- *If you were invited to take the test by your GP, would you have it?*
- *Why do you think you would (not) be willing to have the test?*
    - *Break down different parts of the test process e.g. swallowing the capsule, string, pulling the sponge back up, speed of process, outcome of test*
- *The words 'sponge' and 'string' in the description*
- *The colour of the cytosponge*
- *Do you think other people like you would have it?*
- *Why do you think they would (not) be willing to have the test?*
- *The name of the test*

I am now going to show you a short video about how the cytosponge works…

(Show video of the cytosponge)

- Can you now please tell me your impressions of the test after watching this video?

Prompts:

- *Has it changed your opinion about whether you would you have it?*
- *What are the reasons it has (has not) changed your opinion?*
    - *Break down different parts of the test process e.g. swallowing the capsule, string, pulling the sponge back up, speed of process, outcome of test*
- *Do you think that other people would have it?*
- *Why do you think they would (not) be willing to have the test?*

4) **Best3 trial**

We are planning a trial to see how good the Cytosponge test is at finding Barrett's oesophagus in patients aged 50+ with regular heartburn. People will be invited to join the trial by their GP. The Cytosponge test will be carried out by a nurse in the GP practice or at a local endoscopy centre.

- If you were invited to take part in this trial, what would you want to know about the Cytosponge test before deciding whether or not to take part?

Prompts:

- *Accuracy of the test? (high)*
- *Risks of the test? (no known risks)*
- *Time taken to get results?*
- *Amount of information on link between heartburn, Barrett's Oesophagus and Oesophageal cancer*
- *Type of information (leaflet, video, webpage etc.)*
- *What do you think other people would want to know?*

5) **Endoscopy**

At the moment, in routine clinical care, Barrett's Oesophagus can only be diagnosed by endoscopy. Endoscopy is a procedure where the inside of your body, for example your food pipe (oesophagus) or stomach, is examined using a long, thin, flexible tube that has a light source and a video camera at one end. This is called an endoscope. The endoscope is inserted through the mouth down the oesophagus and images of the inside of the oesophagus can then be seen on a computer screen. This process takes up to an hour and is performed by a gastroenterologist. You can choose to be sedated for the procedure. If you are sedated, you might be so heavily sedated that you will not be aware of the endoscopy going on. You then cannot drive for the rest of the day and someone must supervise you. As this procedure is quite invasive and unpleasant, most patients with heartburn will not be referred to have one.

An endoscopy looks something like this…

(Show picture/ diagram of endoscopy)

- Have you ever had an endoscopy?
- If yes: How did you find the test?
- If no: What are your impressions of this test?

Prompts:

- *Given the option, which test would you prefer to have? Why?*
- *Positives and negatives, e.g. sedation, discomfort, sensitivity of test, risks of procedure, time taken*
- *Which test do you think other people would prefer? Why?*

If the test for Barrett's Oesophagus is positive patients will be sent for an endoscopy to confirm the Cytosponge test. Patients with Barrett's Oesophagus will have an endoscopy every year or two to check for complications and signs of cancer.

- Would the fact that you would have to have regular endoscopies if you tested positive for Barret's oesophagus influence your decision to have the cytosponge test in the first place?
- Would you want to know about his when deciding whether to have the cytosponge test?

6) Cancer Prevention

As I mentioned earlier, unfortunately, we cannot treat Barrett's oesophagus at the moment. A lot of research currently being carried out trying to find ways to prevent cancer in patients with Barrett's oesophagus. So we are hoping that we will soon be able to reduce the chance of oesophageal cancer in patients with Barrett's oesophagus.

- Would knowing about new ways of preventing oesophageal cancer influence your decision whether to have the cytosponge test?

Closing

- Is there anything else you would like to add about heartburn, Barrett's Oesophagus or Oesophageal cancer?
- Is there anything you think we should know from a patient's perspective about doing the trial I described to you?

(Finish recording)

Thank you so much for taking part in this study. If you have any further questions, please feel free to contact me. Here is a debrief sheet for you containing some more information about the study.

(Give debrief sheet)
