# Supplementary material for: Acceptability of the Cytosponge procedure for detecting Barrett's oesophagus: a qualitative study
Source: BMJ Open. 2017 Mar 1;7(3):e013901. doi: 10.1136/bmjopen-2016-013901 (PMC5353314; doi:10.1136/bmjopen-2016-013901)
Supplement: supplementary document [file bmjopen-2016-013901supp2.pdf]

Supplementary Document 2: PowerPoint presentation used in the focus groups.

Heartburn Focus Group

Madeleine Freeman, Sam Smith and Judith Offman

Centre for Cancer Prevention

Queen Mary University of London

Welcome

- Your rights:
  - You may withdraw at any stage
  - Data will be kept confidential
  - Right to be respected by others
- Please try to only discuss with other people when we ask you to.
- However if something is unclear or if you would like it repeated, please ask.

What is Heartburn?

Images of oesophagus and stomach

removed for copyright purposes

- A burning symptom in your chest and throat. It is sometimes called acid reflux.
- Heartburn is caused by stomach acid splashing up from the stomach into the oesophagus (food pipe).
- Very common – 1 in 5 adults experience it each week.

- Can you please speak a bit about your experiences of heartburn?

Illustration of heartburn

removed for copyright purposes

Heartburn Treatment

- Heartburn can usually be treated with medication and lifestyle changes, for example avoiding spicy foods and avoiding eating heavy meals late at night.

Illustrations of a curry and heartburn medication

removed for copyright purposes

Barrett's Oesophagus

- Some people who have regular heartburn develop a condition called Barrett's Oesophagus.
- Has anybody heard of this condition before?

Supplementary Document 2: PowerPoint presentation used in the focus groups.

Barrett's Oesophagus

- This is when the cells in the food pipe change shape from flat to rectangular.
- People with Barrett's Oesophagus can experience long-term indigestion, swallowing can become difficult and food can come back up from the stomach into the oesophagus.
- About 4 in 100 people with regular heartburn develop Barrett's Oesophagus

Images of gastro-oesophageal junction with and without Barrett's Oesophagus

removed for copyright purposes

Risk Factors for Barrett's Oesophagus

- Genetic predisposition
- Male sex
- Caucasian ethnicity
- Aged over 50
- Overweight
- Smoking
- Drinking alcohol
- Eating fatty and spicy foods

Images of a pint of beer, place of chips and a cigarette

removed for copyright purposes

What does it mean if a person has Barrett's Oesophagus?

- A very small number of people with Barrett's Oesophagus develop Oesophageal cancer (around 1 in 200 people).
- Oesophageal cancer is the 11th most common cancer in the UK.
- Most cases of oesophageal cancer are diagnosed late, where treatment is more difficult.
- If we know that somebody has Barrett's Oesophagus we can monitor them regularly to look for abnormalities and provide immediate treatment if oesophageal cancer is detected.

How can we detect Barrett's Oesophagus?

- Scientists at the University of Cambridge have developed a new test to identify Barrett's Oesophagus.
- It is called the Cytosponge, and looks like this…

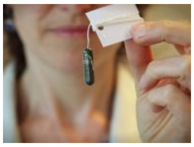

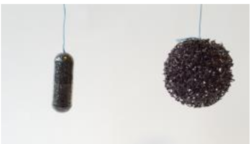

The Cytosponge

- The Cytosponge is a small, 2cm capsule on a string which you swallow. After the Cytosponge has been swallowed, it sits in the stomach for three or four minutes and expands.
- The sponge is then pulled back up out of your mouth using the string attached. The whole process takes about five minutes and can be carried out by a nurse or a doctor.
- The sponge picks up about half a million tiny cells from your oesophagus and these are sent to the laboratory to look for signs of Barrett's Oesophagus.

The Cytosponge

- What are your impressions of the test after hearing this information?

Supplementary Document 2: PowerPoint presentation used in the focus groups.

The Cytosponge Samples

- Here are some samples of the Cytosponge
- Please have a look at them and pass them around

The Cytosponge Video

- You are now going to watch a short video about how the Cytosponge works:

https://www.youtube.com/watch?v=s7X9z6qlNUI

BEST3 Trial

- We are planning a trial to see how good the Cytosponge is at finding Barrett's oesophagus in patients over 50 with regular heartburn.
- People will be invited to join the trial by their GP.
- The Cytosponge test will be carried out by a nurse in the GP practice or at a local endoscopy centre.

Illustration of GP surgery

removed for copyright purposes

BEST3 Trial

- If you were offered, would you be interested in taking part in this trial?

BEST3 Trial

- If you were invited to take part in this trial, what would you want to know about the Cytosponge test before deciding whether or not to take part?

BEST3 Trial

- How much information would you want to be given on the link between heartburn, Barrett's Oesophagus and Oesophageal cancer?

Supplementary Document 2: PowerPoint presentation used in the focus groups.

BEST3 Trial

- What type of information would you prefer to be given if you were invited to take part?

Images of a leaflet, film camera and web-page

removed for copyright purposes

Current Practice

- At the moment in routine clinical care, Barrett's Oesophagus can only be diagnosed by endoscopy.

- What do you know about endoscopies?

Endoscopy

- An endoscopy looks something like this…

Illustration of an endoscopy procedure

removed for copyright purposes

Endoscopy

- What are your impressions of endoscopy after hearing and seeing this information?

Cytosponge vs. Endoscopy

- Given the option, which of these two tests would **you** prefer to have and why?

Cytosponge vs. Endoscopy

- Which of the two tests do you think **other people** would prefer to have and why?

Supplementary Document 2: PowerPoint presentation used in the focus groups.

Positive Cytosponge Test

- If the Cytosponge test is positive, and so Barrett's Oesophagus is detected, patients will be sent for an endoscopy to confirm this.
- Patients with Barrett's Oesophagus will have an endoscopy every year or two to check for complications and signs of cancer.

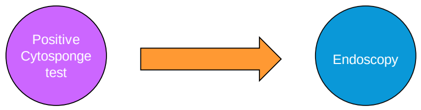

Final comments

- Is there anything else you would like to add about heartburn, Barrett's Oesophagus or Oesophageal cancer?

Thank you for your time

If you have any more questions please do not hesitate to contact me:

m.freeman@qmul.ac.uk
